# Constituents and Pharmacological Activities of *Myrcia* (Myrtaceae): A Review of an Aromatic and Medicinal Group of Plants

**DOI:** 10.3390/ijms161023881

**Published:** 2015-10-09

**Authors:** Márcia Moraes Cascaes, Giselle Maria Skelding Pinheiro Guilhon, Eloisa Helena de Aguiar Andrade, Maria das Graças Bichara Zoghbi, Lourivaldo da Silva Santos

**Affiliations:** 1 Programa de Pós-graduação em Química, Universidade Federal do Pará, Belém 66075-110, PA, Brazil; E-Mails: cascaesmm@gmail.com (M.M.C.); eloisandrade@ufpa.br (E.H.A.A.); lss@ufpa.br (L.S.S.); 2 Museu Paraense Emílio Goeldi, Belém 66040-170, PA, Brazil; E-Mail: gracazoghbi@gmail.com

**Keywords:** *Myrcia*, volatiles, non-volatiles, biological activities

## Abstract

*Myrcia* is one of the largest genera of the economically important family Myrtaceae. Some of the species are used in folk medicine, such as a group known as “pedra-hume-caá” or “pedra-ume-caá” or “insulina vegetal” (insulin plant) that it is used for the treatment of diabetes. The species are an important source of essential oils, and most of the chemical studies on *Myrcia* describe the chemical composition of the essential oils, in which mono- and sesquiterpenes are predominant. The non-volatile compounds isolated from *Myrcia* are usually flavonoids, tannins, acetophenone derivatives and triterpenes. Anti-inflammatory, antinociceptive, antioxidant, antimicrobial activities have been described to *Myrcia* essential oils, while hypoglycemic, anti-hemorrhagic and antioxidant activities were attributed to the extracts. Flavonoid glucosides and acetophenone derivatives showed aldose reductase and α-glucosidase inhibition, and could explain the traditional use of *Myrcia* species to treat diabetes. Antimicrobial and anti-inflammatory are some of the activities observed for other isolated compounds from *Myrcia*.

## 1. Introduction

*Myrtaceae Juss*. is the ninth largest flowering plant family; it includes trees and shrubs with centers of diversity in the wet tropics, particularly in South America, Australia and Tropical Asia, distributed in 132 genera and 5671 species [1,2]. It also represents one of the largest families of the Brazilian flora, where 23 genera and 1034 species occur distributed in all regions and vegetal formations of the country [1,2,3,4]. Economically, Myrtaceae is a very important family; some species are cultivated, such as *Eucalyptus* spp., from which the wood is used to produce paper, lamppost and charcoal; other species are ornamental and some are used as spices, such as *Syzygium aromaticum* (L.) Merr. & L.M.Perry, known as “*cravo-da-índia*” or clove. Several species produce edible fruits that are used to make juice, jelly and sweets, such as *Psidium guajava* L. (“goiabeira” and “guava”), *Myrciaria cauliflora* (Mart.) O.Berg (“jabuticabeira”), *Eugenia uniflora* L. (“pitangueira”), *Syzygium* spp. (“jambo”), but not all are cultivated. Myrtaceae species are also used in folk medicine to treat several diseases, especially gastrointestinal disorders, hemorrhagic and infectious diseases with an action that is probably related to its astringents properties [5].

*Myrcia sensu lato* or *Myrcia* s.l. (*sensu* Lucas *et al.*, 2011) is now considered a large genus taken in a loose sense, composed of four traditional genera (*Myrcia* DC., *Marlierea* Cambess, *Calyptranthes* Sw*.* and *Gomidesia* O.Berg), comprising 753 species [1], that are now in process of synonimization based on recent molecular findings [6,7,8]. According to Rosario and coworkers, many species of these four genera found in the Amazon still need phylogenetic analysis [9].

There are 260 listed *Myrcia* species in Brazil found in different biomes and in all five regions of the country [2]. *Myrcia* species are an important source of essential oils and some species have been used in folk medicine. In the present work, traditional uses, identified volatile and non-volatile compounds, and the pharmacological activities of crude extracts, essential oils and isolated compounds from *Myrcia sensu stricto* are reviewed.

## 2. Traditional Uses

Some *Myrcia* species have been used in folk medicine, usually as infusions, for a long time [10,11,12,13]. The most cited traditional use of *Myrcia* species is related to a small group of Myrtaceae known in Brazil as “pedra-hume-caá”, “pedra-ume-caá” or “insulina vegetal” (insulin plant). The leaves or whole plant infusions of these plants are used to treat diabetes; this group of plants includes *Myrcia* (*M.*) *punicifolia* (Kunth) DC., *M. speciosa* (Amsh.) Mc Vaugh, *M. amazonica* DC., *M. citrifolia* (Aubl.) Urb., *M. guianensis* (Aubl.) DC., *M. multiflora* (Lam.) DC., *M. salicifolia* DC., *M. sylvatica* (G. Mey) DC., *M. uniflora* DC. besides *Eugenia punicifolia* (Kunth) DC. [14,15]. *Myrcia uniflora* is sold as a dry extract in capsules or as tinctures for the treatment of diabetes. “Pedra-ume-caá” is also used to treat diarrhea, enteritis, hemorrhage and aphtha [10] and *M. salicifolia* is good for cold sores and mouth ulcers [16]. Other *Myrcia* species have traditional uses: *Myrcia bracteata* DC. is used to treat dyspepsia [13]; *M. ovata* Cambess. is used on the treatment of gastric illness, gastritis and diarrhea [17]; and the inhabitants of the Amazon region use the macerated leaves of *M. guianensis* to neutralize snake venoms [18].

## 3. Volatiles

Most chemical and biological studies on *Myrcia* deal with the essential oils obtained from these species [19]. Major compounds from the essential oils of *Myrcia* (>5%) are summarized in Table 1, together with the classes and the total of the identified compounds, in accordance to literature.

**Table 1:** Relative abundance (>5%) of the constituents in the essential oils from *Myrcia* species.

Species	Part of the Plant (Yield: *v*/*w*)	Compounds (Relative Abundance, %>5) Classes of Substances; Total	Ref.
*M. acuminatissima* O.Berg	Fresh leaves (0.12%)	β-pinene (5.0), linalool (22.3), terpinen-4-ol (5.2), β-caryophyllene (8.1), spathulenol (7.5), caryophyllene oxide (5.5) MH: 13.9%, OM: 35.1%, SH: 15.7%, OS: 28.0%, T: 97.0%	[20]
*M. alagoensis* O.Berg	Fresh leaves (0.3%)	β-caryophyllene (7.9), germacrene D (11.1), germacrene B (26.7), (2*E*,6*E*)-farnesoic acid (7.3) S: 79.9%, T: 80.5%	[21]
	Dry leaves (0.4%)	β-caryophyllene (7.8), germacrene D (6.4), δ-cadinene (5.4), selina-3,7(11)-diene (5.4), germacrene B (23.1) S: 75.4%, T: 75.5%	[21]
*M. amazonica* DC.	Fresh leaves (0.65%)	germacrene D (10.09), germacrene B (9.59), 1-*epi*-cubenol (20.22), α-muurolol (6.21)	[22]
	Dry leaves (0.96%)	germacrene D (16.56), germacrene B (11.09), 1-*epi*-cubenol (14.72)	[22]
*M. arborescens* O.Berg	Fresh leaves (0.2%)	α-muurolol (6.2), caryophyllene oxide (26.3), spathulenol (8.9), globulol (15.9), 5-*epi*-7-*epi*-α-eudesmol (5.9) S: 94.8%, T: 96.2%	[23]
*M. bombycina* (O.Berg) Nied.	Fresh leaves (0.95%)	α-pinene (23.9), β-pinene (12.4), limonene (7.0), γ-eudesmol (7.8) MH: 53.0%, OM: 5.4%, SH: 8.0%, OS: 24.0%, T: 93.5%	[20]
*M. bracteata* DC.	Leaves (0.71%)	α-bisabolol oxide (10.37), α-bisabolol (45.86) SH: 13.76%, OS: 63.49%, S: 77.25%	[24]
	Leaves and fine stems (0.1%) ^a^	(*E*)-nerolidol (80.8)	[25]
	Leaves and fine stems (0.3%) ^a^	(*E*)-β-farnesene (33.9), β-curcumene (9.8), β-bisabolol (8.2)	[25]
	Leaves and fine stems (0.1%) ^a^	germacrene B (8.8), spathulenol (31.0)	[25]
*M. cuprea* (O.Berg) Kiaersk.	Leaves (<0.05%)	β-caryophyllene (9.57), α-humulene (7.03), γ-selinene (21.75), α-selinene (11.84), (*Z*)-α-bisabolene (9.51), (*E*,*E*)-α-farnesene (10.52) SH: 82.41%, S: 82.41%, OTH: 11.02%	[24]
	Leaves and fine stems (0.3%) ^a^	myrcene (48.1), β-caryophyllene (19.9), δ-cadinene (6.9)	[25]
	Leaves and fine stems (0.1%) ^a^	α-pinene (15.9), myrcene (19.2), β-caryophyllene (39.1)	[25]
	Leaves and fine stems (>0.1%) ^a^	β-caryophyllene (38.1), germacrene D (21.8), germacrene B (19.5)	[25]
*M. fallax* (Rich.) DC.	Leaves (0.09%)	α-pinene (7.68), β-pinene (11.88), β-elemene (11.21), β-caryophyllene (5.55), selin-11-en-4α-ol (7.56) MH: 22.29%, M: 24.02%, SH: 41.83%, OS: 24.20%, S: 66.03%	[24]
	Leaves (0.25%)	α-pinene (7.7), β-pinene (6.9), β-caryophyllene (6.0), carotol (9.9), guaiol (31.0) T: 83.4%	[26]
	Flowers (0.30%)	α-pinene (6.0), guaiol (27.5), aristolene (24.5) T: 83.2%	[26]
	Fresh leaves (0.25%)	α-bisabolol (83.8) SH: 7.2%, OS: 86.5%, T: 94.3%	[20]
*M.* aff. *fosteri* Croat	Leaves	β-bisabolol oxide (19.2), α-bisabolol (19.2), bisabolol oxide B (7.0), undeca-4,6-diene (5.4) SH: 8.2%, OS: 65.9%, T: 76.7%	[27]
*M. glabra* (O.Berg) D.Legrand	Fresh leaves (0.11%)	α-copaene (6.1), β-caryophyllene (9.5), β-selinene (5.8), α-selinene (9.4), valerianol (13.2) SH: 54.4%, OS: 27.4%, OTH: 9.4%, T: 92.0%	[20]
*M. hatschbachii* D.Legrand	Fresh leaves (0.1%)	germacrene D (6.4%), γ-cadinene (8.1), α-cadinol (6.1), β-caryophyllene (23.3) S: 95.2%, T: 97.9%	[23]
*M. lageana* D.Legrand	Fresh leaves (0.3%)	(*E*)-nerolidyl acetate (25.3), germacrene D (23.4) S: 98.5%, T: 99.3%	[23]
*M. laruotteana* Camb.	Unripe fruits (0.3%)	spathulenol (5.4), globulol (6.3), α-bisabolol oxide B (11.5), α-bisabolol (23.6), globulol (6.3), (2*E*,6*E*)-methyl farnesoate (5.8) SH: 5.8%, OS: 75.8%, T: 82.8%	[28]
	Leaves (0.05%)	spathulenol (7.3), globulol (6.2), guaiol (6.1), 1-*epi*-cubenol (5.0), α-cadinol (8.0), α-bisabolol (20.7), 14-hydroxy-α-muurolene (19.9) SH: 10.2%, OS: 79.6%, T: 90.4%	[29]
	Flowers (0.07%)	spathulenol (8.6), globulol (6.6), guaiol (7.7), 1-*epi*-cubenol (5.0), α-cadinol (6.5), α-bisabolol (28.1), 14-hydroxy-α-muurolene (13.7) SH: 9.1%, OS: 83.1%, T: 95.5%	[29]
*M. multiflora* (Lam) DC.	Leaves (1.16%)	α-gurjunene (6.40), β-caryophyllene (10.72), γ-selinene (5.12), α-selinene (8.67), selin-11-en-4α-ol (10.67) MH: 6.14%, OS: 5.23%, M: 11.37%, SH: 53.91%, OS: 17.57%, S: 71.48%	[24]
	Fresh leaves (0.20%)	β-caryophyllene (7.5), germacrene D (8.7), bicyclogermacrene (6.3), δ-cadinene (5.2), MW 222 (7.4), cubenol (5.9) SH: 44.9%, OS: 32.3%, n.i.: 5.9%, T: 87.5%	[20]
*M. myrtillifolia* DC.	Leaves (0.14%)^b^	α-pinene (80.4), α-terpineol (7.0) MH: 85.4%, OM: 13.0%, T: 99.7%	[30]
	Flowers (0.26%) ^b^	α-pinene (76.2) MH: 77.1%, OM: 9.9%, T: 88.8%	[30]
	Fruits (0.37%) ^b^	α-pinene (88.1) MH: 91.9%, T: 96.8%	[30]
*M. obtecta* (O.Berg) Kiaersk.	Fresh leaves (0.1%)	α-pinene (7.2), ar-curcumene (19.0), β-bisabolene (8.5), α-copaene (8.0), α-humulene (6.2) M: 16.2%, S: 79.1%, T: 95.3%	[23]
*M. obtecta* (O.Berg) Kiaersk.	Leaves (0.01%) ^b^	α-terpineol (11.2), α-guainene (5.8), *trans*-calamenene (29.3), 1-*epi*-cubenol (5.6) M: 16.7%, SH: 56.4%, OS: 20.9%, T: 95.6%	[31]
	Flowers (n.i.)	methyl salicilate (88.2) T: 97.9%	[31]
*M. oligantha* O.Berg	Fresh leaves (0.1%)	δ-cadinene (17.9), 1-*epi*-cubenol (7.2), cubenol (5.7), β-caryophyllene (6.5), caryophyllene oxide (5.4), bicyclogermacrene (8.3), spathulenol (10.2) S: 96.8%, T: 99.9%	[23]
*M. ovata* Cambess.	Leaves (0.9%)	neral (35.8), geranial (50.4) T: 92.1%	[17,32]
	Leaves (1.27%; *w*/*w*)	OM: 91.78%, T: 93.55%	[33]
*M. pubiflora* DC.	Fresh leaves (1.1%)	tricyclene (5.27), 1,8-cineole (5.35), caryophyllene oxide (22.16), mustakone (11.34) T: 72.7%	[34]
*M. pubipetala* Miq.	Fresh leaves (0.1%)	germacrene D (7.2), β-caryophyllene (13.3), bicyclogermacrene (25.2), spathulenol (31.7), *n*-heneicosane (14.9) OTH: 14.9%, S: 84.8%, T: 99.7%	[23]
*M. richardiana* (O.Berg) Kiaersk.	Fresh leaves (0.1%)	β-caryophyllene (20.6), caryophyllene oxide (19.3), α-humulene (5.1), bicyclogermacrene (5.7) S: 90.0%, T: 90.0%	[23]
*M. rostrata* DC.	Fresh leaves (0.2%)	δ-cadinene (5.7), τ-muurolol (5.1), caryophyllene oxide (13.1), bicyclogermacrene (6.8), spathulenol (17.3) S: 93.3%, T: 93.3%	[23]
*M. rufipila* McVaugh	Leaves (0.42%) ^a^	β-caryophyllene (7.07), γ-elemene (10.49), germacrene D (9.09), bicyclogermacrene (7.49), δ-cadinene (7.36), germacrene B (6.70) SH: 72.35%, OS: 19.93%, S: 92.28%	[24]
	Leaves (0.18%) ^a^	β-caryophyllene (5.66), germacrene D (10.31), δ-cadinene (10.12), α-cadinol (6.20) SH: 65.87%, OS: 23.69%, S: 89.56%	[24]
*M. salzmanni* O.Berg	Leaves (n.i.) ^b^	β-caryophyllene (25.9), α-humulene(12.9), MW 222 (11.7), MW 220 (14.2), MW 222 (10.0) SH: 49.2%, OS: 10.1%, NI: 36.2%, T: 95.5%	[35]
	Flowers (n.i.)	β-caryophyllene (13.8), α-humulene (10.9), MW 222 (10.0), MW 220 (12.6), *cis*-β-elemone (6.2), MW 222 (7.1) SH: 36.4%, OS: 20.1%, NI: 38.6%, T: 95.4%	[35]
*M. selloii* (Spreng.) N.Silveira	Fresh leaves (0.5%)	germacrene D (6.7), δ-cadinene (14.5), τ-cadinol (9.3), α-cadinol (17.2), β-caryophyllene (9.0), bicyclogermacrene (10.2) S: 99.2%, T: 99.9%	[23]
*M. splendens* (Sw.) DC.	Fresh leaves (0.44%)	(*Z*)-α-bisabolene (79.65) SH: 94.54%, S: 98.34%, T: 98.34%	[36]
	Fresh stems (0.15%)	β-caryophyllene (23.8), germacrene D (25.3), bicyclogermacrene (7.1), caryophyllene oxide (10.5) T: 97.2%	[37]
	Leaves (n.i.)	*trans*-2-hexenal (9.5), germacrene D (35.9), δ-cadinene (5.8), *epi*-α-cadinol (6.8), valerianol (16.3) SH: 55.7%, OS: 31.8%, T: 96.9%	[38]
*M. sylvatica* (G.Mey) DC.	Leaves and fine stems (>0.1%) ^a^	spathulenol (13.8), caryophyllene oxide (16.6), selin-11-en-4α-ol (24.7)	[25]
	Leaves and fine stems (0.3%) ^a^	*cis*-calamenene (30.1), α-calacorene (11.5), spathulenol (18.7)	[25]
	Leaves and fine stems (>0.1%) ^a^	β-bisabolene (14.7), spathulenol (40.2)	[25]
*M. tomentosa* (Aubl.) DC.	Aerial parts (0.54%) ^b^	(*E*)-β-farnesene (6.94), γ-muurolene (18.04), bicyclogermacrene (11.51), (2*E*,6*E*)-methyl farnesoate (36.95) SH: 47.22%, OS: 52.02%, T: 99.24%	[39]
	Fresh flowers (0.31%)	spathulenol (7.36), globulol (5.97), (2*Z*,6*Z*)-farnesal (6.86), (2*Z*,6*Z*)-farnesol (10.65), (2*E*,6*E*)-farnesal (5.36), (2*E*,6*E*)-methyl farnesoate (14.28), benzyl salicylate (5.99) OS: 46.69%, OTH: 24.61%, T: 72.71%	[39]
	Stem bark (0.31%)	(2*E*,6*E*)-methyl farnesoate (14.39), hexadecanoic acid (22.05) SH: 6.26%, OS: 9.06%, OTH: 60.4%, T: 76.27%	[39]
	Leaves (0.1–0.8) ^b^	spathulenol (18.35), globulol (7.66), (2*E*,6*E*)-methyl farnesoate (46.38)	[40]
	Leaves (0.1–0.8) ^b^	γ-muurolene (14.20), bicyclogermacrene (14.38), δ-amorphene (18.83)	[40]
	Leaves (0.1–0.8) ^b^	γ-muurolene (6.62), bicyclogermacrene (8.04), globulol (57.48)	[40]
	Leaves (0.1–0.8) ^b^	γ-muurolene (7.67), bicyclogermacrene (5.85), (2*E*,6*E*)-methyl farnesoate (60.69)	[40]
	Leaves (0.1–0.8) ^b^	β-caryophyllene (12.66), γ-muurolene (40.16), bicyclogermacrene (13.74), δ-amorphene (6.31)	[40]

M: Monoterpenes (hydrocarbons and oxygenated); S: Sesquiterpenes (hydrocarbons and oxygenated); MH: Monoterpene Hydrocarbons; OM: Oxigenated Monoterpenes; SH: Sesquiterpene Hydrocarbons; OS: Oxigenated Sesquiterpenes; OTH: Others; NI: not identified; T: total of the identified compounds; ^a^ Different site of collection for a same species in a same reference; ^b^ Studies of seasonal or circadian variations (oils with the highest T and measured yield is listed); n.i.: not informed; Ref.: Reference.

Leaves, flowers, stems, fruits of *Myrcia* can produce essential oils. Sesquiterpenes are the major compounds in most of these oils, although monoterpenes were identified in a higher amount than sesquiterpenes in the essential oil of *M. acuminatissima* and *M. bombycina* [20], one of the studied specimen of *M. cuprea* [25], *M. myrtillifolia* [30] and *M. ovata* [17,32,33]. The major compound of the essential oil of *M. obtecta* flowers of was methyl salicylate [31], and the most abundant compound of the essential oil of *M. tomentosa* stem bark was decanoic acid [39].

According to Alarcón and coworkers [26], the essential oils of *M. fallax* collected in Venezuela were different; leaves and flowers oils were rich on guaiol/carotol and guaiol/aritolone, respectively. Additionally, it was observed that the essential oils from *M. falax* collected in Venezuela were also different from the specimens from Brazil, in which those terpenes were not identified [20,24].

Siani and coworkers arranged the mono- and sesquiterpenes from 15 Neotropical Myrtaceae in accordance to their biosynthetic pathways; the species showed a heterogeneous composition, with a wide variation with respect to terpenoid structures, including bisabolene-type; no chemotaxonomical implications were found [41].

Seasonal variation studies have demonstrated that the essential oil of *M. obtecta* leaves did not exhibitet important differences on the composition, except for the flowering month, when α-terpineol and *trans*-calamenene were detected on the highest amounts [31]. The essential oil of the leaves of *M. tomentosa* exhibited seasonal variation; it was observed that only nine of 44 compounds were identified in all samples, indicating a significant correlation between the climatic data, foliar nutrients and essential oil composition [39]. Cluster and Principal Component analysis indicated a high chemovariability within the essential oils of *M. tomentosa* [39]. The oil from *M. salzmannii* leaves showed qualitative and quantitative variations in the composition; only two compounds, β-caryophyllene and α-humulene, were identified in all samples [35]. According to Zoghbi and coworkers, the essential oils of *M. sylvatica* show intraspecific variation [25].

## 4. Biological and Antioxidant Activities of the Essential Oils of *Myrcia* Species and Their Major Constituents

Several studies have shown the biological activities of *Myrcia* essential oils [19]. The number of published papers is growing every day.

### 4.1. Anti-Inflammatory and Antinociceptive Effect

Essential oils from *M. ovata* leaves (50–300 mg/kg of oral doses) showed significant effect in acute pain and inflammation tests with no adverse effects and intoxication during the assays; according to the authors, these results provided initial evidence of the traditional use of this species [33].

The essential oil from the fresh leaves of *M. pubiflora* (25, 50 and 100 mg/kg) significantly reduced the number of writhing induced by acetic acid and the nociception in the second phase of formalin test; it exhibited inhibitory effect on carrageenan-induced response, it was ineffective inhibiting the time for reaction to thermal stimulus and it did not show any motor performance alterations [34].

### 4.2. Antimicrobial Activity

The essential oil of *M. ovata* leaves shows antimicrobial action against several microorganisms [13]. The studies of Alarcón and coworkers [26] showed that the essential oil of *M. fallax* flowers from Venezuela is active only against the Gram positive bacteria and not against the Gram negative bacteria. The oil from *M.* aff. *fosteri* showed activity against two bacteria which was comparable to chloramphenicol [27]. The leaves essential oils of *M. myrtillifolia* showed antimicrobial activity against several microorganisms, with a moderate toxicity against *Artemia salina* [30]. The essential oils of *M. alagoensis* exhibited a broad spectrum of antibacterial action, on both Gram positive and Gram negative bacteria, and the former were more sensitive to the essential oil from the fresh leaves [21]. Antibacterial activity was observed when essential oil from the stems of *M. splendens* was tested [37].

### 4.3. Larvicidal Activity

The essential oil of *M. ovata* leaves, which is rich in citral (neral: 35.8%; geranial: 50.4%), showed larvicidal activity against *Aedes aegypti* [32]. According to these authors the essential and their major compounds may be potent source of natural larvicides.

### 4.4. Antiproliferative Activity

The essential oil from *M. laruotteana* fruits and the fraction rich in α-bisabolol when tested against *in vivo* human cancer cells (glioma, melanoma, breast, ovarian and ovarian-resistant, kidney, lung, prostate, colon and leukemia) showed antiproliferative activity against all cell lines, except for the lung cell line; the α-bisabolol rich fraction had a similar profile [28].

### 4.5. Antioxidant Capacity

The essential oil from *M. amazonica* leaves showed a higher antioxidant activity than BHT (buthyl-hydroxytoluene) and α-tocopherol when using the ORAC (oxygen radical absorbance capacity) method, but lower when the cation-radical ABTS (2,2ʹ-azino-bis(3-ethylbenzthiazoline-6-sulfonic acid)) method was applied, using TROLOX as reference [22].

## 5. Activity of the Major Compounds from *Myrcia* Essential Oils

Some of the constituents of *Myrcia* essential oils show activities that could contribute to the biological properties, however, in all cases synergistic and antagonist influence of the various components should be considered.

Citral has a significant central and peripheral antinociceptive effect and anti-inflammatory activity [42]. In that way, citral, the major component of *M. ovata* essential oil, can contribute for the observed analgesic activity of the essential oil.

The sesquiterpene caryophyllene oxide exhibits antinociceptive activity [43]; the monoterpene 1,8-cineole also shows antinociceptive properties on hot plate and tail-flick tests, while β-pinene exerts supraspinal and antinociceptive actions in rats, but it reverses the effect of morphine [44]. These compounds were identified in several essential oils from *Myrcia*.

Terpinen-4-ol, linalool, α-terpineol and β-caryophyllene are known for their antimicrobial activities [45,46]. These compounds probably contribute to some of the observed activities.

## 6. Non-Volatiles

The chemical studies regarding to the non-volatile compounds identified from *Myrcia* species mostly describe the isolation of flavonol glucosides. Together with flavonoids, terpenoids, organic acids, acetophenones and related compounds have been isolated. A small number of species have been studied for their chemical composition on non-volatile compounds.

### 6.1. Flavonoids

**Figure 1:**
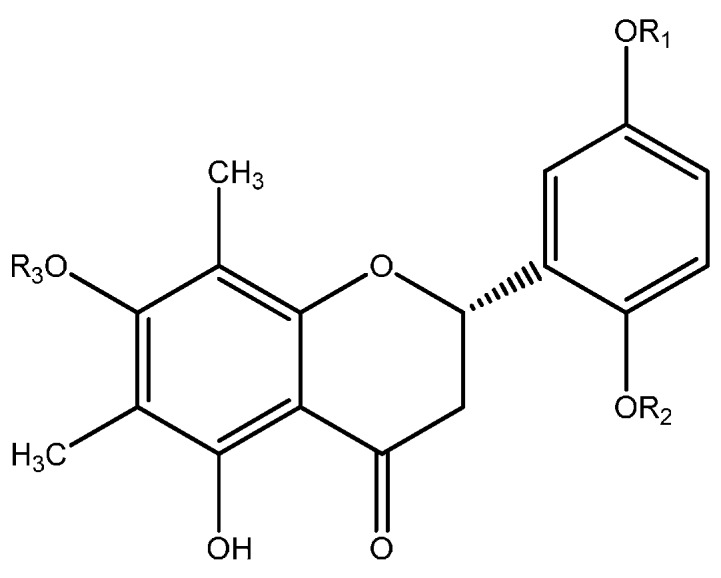
Structures of the flavanone glucosides Myrciacitrins.

**Figure 2:**
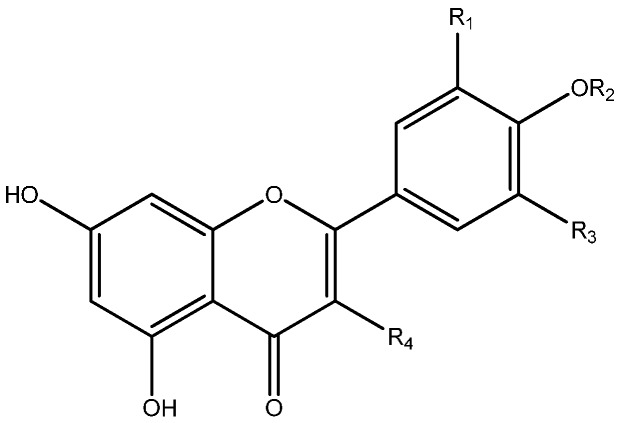
Structures of the flavonols and their glucosides isolated from *Myrcia*.

**Figure 3:**
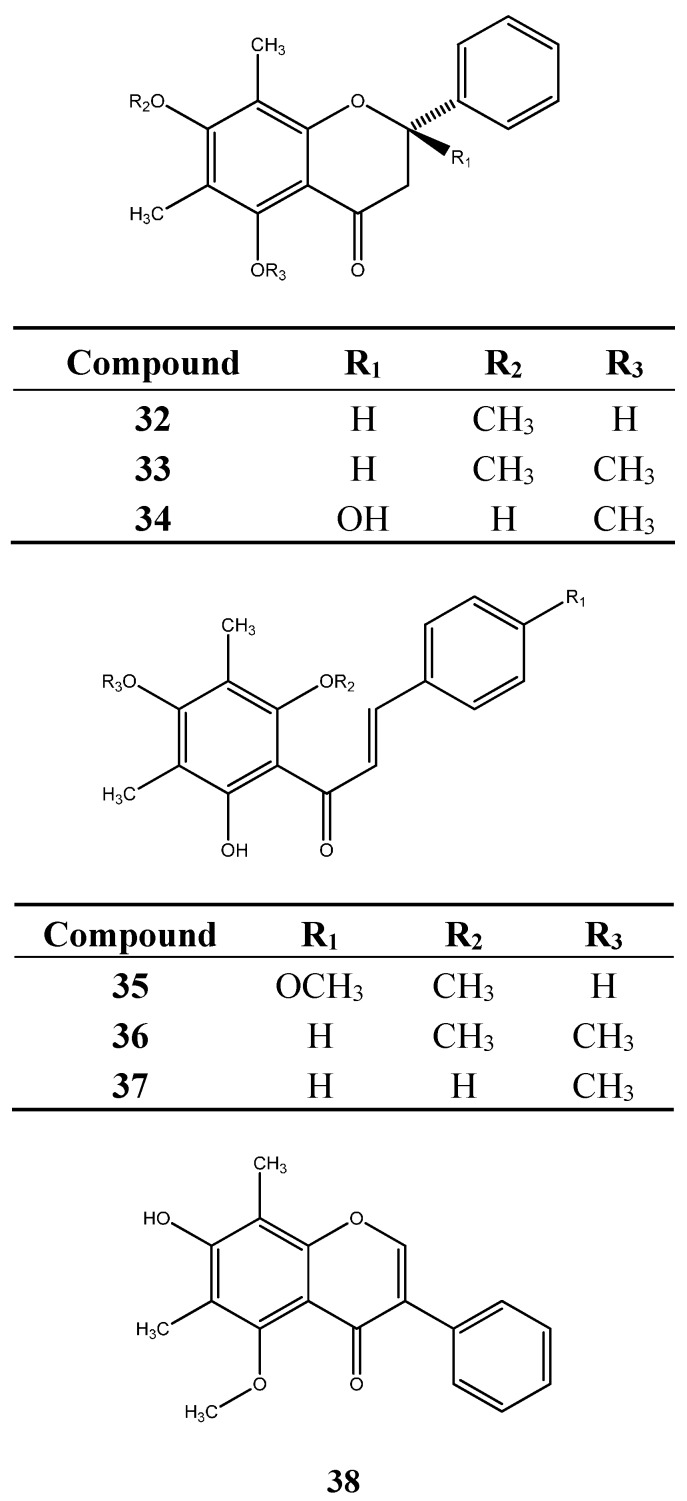
Other flavonoids and derivatives isolated from *Myrcia* species.

The flavonoids isolated from *Myrcia* are mostly flavanones and flavonol-*O*-glycosides. The sugar units are usually galactose, glucose, xylose and rhamnose. Structures of the isolated flavonoids from *Myrcia* are in Figure 1, Figure 2 and Figure 3.

The extracts of *M. multiflora* leaves contain the flavanone glucosides myrciacitrins I (**1**), II (**2**), III (**3**), IV (**4**) and V (**5**) [47,48]. To date, the flavanone glucosides have been isolated only from *M. multiflora*.

**Table:** 

Compound	R_1_	R_2_	R_3_
**1**	H	H	*O*-β-d-glucopyranosyl
**2**	CH_3_	H	*O*-β-d-glucopyranosyl
**3**	H	*O*-β-d-glucopyranosyl	H
**4**	H	H	(6ʹʹ-*O*-*p-*coumaroyl)-*O*-β-d-glucopyranosyl
**5**	H	H	(6ʹʹ-*O*-*p*-hydroxybenzoyl)-*O*-β-d-glucopyranosyl

*Myrcia multiflora* extracts also contain the flavonol glucosides myricitrin (**6**), mearnsitrin (**7**), quercitrin (**8**), desmanthin-1 (**9**), guaijaverin (**10**) [47,48]. Myricitrin (**6**) was also isolated from *M. bella* Cambess. [49], *M. splendens* [50], *M. palustris* DC. [50] and *M. uniflora* [51], mearnsitrin (**7**) was also obtained from *M. uniflora* [52]. From the leaves of *M. tomentosa*, avicularin (**11**) and juglanin (**12**) were isolated [53]. Other flavonoids have been isolated from *M. bella*, such as myricetin (**13**), kaempferol-3-*O*-deoxyhexoside (**14**), kaempferol-3-*O*-hexoside (**15**), myricetin-3-*O*-β-d-galactopyranoside (**16**), myricetin-3-*O*-α-arabinofuranoside (**17**), myricetin-3-*O*-α-arabinopyranoside (**18**), myricetin-3-*O*-(*O*-galloyl)-hexoside (**19**), quercetin (**20**), quercetin-3-*O*-β-d-galactopyranoside (**21**), quercetin-3-*O*-β-d-xylofuranoside (**22**), quercetin-3-*O*-β-d-xylopyranoside (**23**), quercetin-3-*O*-α-l-arabinofuranoside (**24**), quercetin-3-*O*-(6ʹʹ-galloyl)-β-galactopyranoside (**25**), quercetin-3-*O*-(*O*-galloyl)-pentoside (**26**) [49]. *Myrcia palustris* also produced desmanthin-1 (**9**), myricetin (**13**), myricetin-3-*O*-β-d-galactopyranoside (**16**), quercetin (**20**), quercetin-3-*O*-β-d-xylopyranoside (**23**) and quercetin-3-*O*-α-l-arabinofuranoside (**24**) together with myricetin-(6ʹʹ-galloyl)-3-*O*-β-d-galactopyranoside (**27**), myricetin-3-*O*-β-d-xylopyranoside (**28**), quercetin-3-*O*-α-l-arabinopyranoside (**29**), quercetin-3-*O*-α-l-rhamnopyranoside (**30**) and kaempferol-3-*O*-β-d-galactopyranoside (**31**) [54]. Quercetin (**20**) was also isolated from *M. myrtillifolia* [55]. Flavonol glucoside is the major class of non-volatile secondary metabolites identified from *Myrcia* species.

**Table:** 

Compound	R_1_	R_2_	R_3_	R_4_
**6**	OH	H	OH	*O*-α-l-rhamnopyranosyl
**7**	OH	CH_3_	OH	*O*-α-l-rhamnopyranosyl
**8**	OH	H	OH	*O*-α-l-rhamnopyranosyl
**9**	OH	H	OH	(2ʹʹ-*O*-galloyl)-*O*-α-l-rhamnopiranosyl
**10**	OH	H	H	*O*-α-l-arabinopyranosyl
**11**	OH	H	H	*O*-α-l-arabinofuranosyl
**12**	H	H	H	*O*-α-l-arabinofuranosyl
**13**	OH	H	OH	OH
**14**	H	H	H	*O*-Deoxyhexosyl
**15**	H	H	H	O-Hexosyl
**16**	OH	H	OH	*O*-β-d-galactopyranosyl
**17**	OH	H	OH	*O*-α-arabinofuranosyl
**18**	OH	H	OH	*O*-α-arabinopyranosyl
**19**	OH	H	OH	(*O*-galloyl)-*O*-hexosyl
**20**	H	H	OH	OH
**21**	H	H	OH	*O*-β-d-galactopyranosyl
**22**	H	H	OH	*O*-β-d-xylofuranosyl
**23**	H	H	OH	*O*-β-d-xylopyranosyl
**24**	H	H	OH	*O*-α-l-arabinofuranosyl
**25**	H	H	OH	(6ʹʹ-*O*-galloyl)-*O*-β-galactopyranosyl
**26**	H	H	OH	(*O*-galloyl)-pentosyl
**27**	OH	H	OH	(6ʹʹ-*O*-galloyl)-*O*-β-d-galactopyranosyl
**28**	OH	H	OH	*O*-β-d-xylopyranosyl
**29**	H	H	OH	*O*-α-l-arabinopyranosyl
**30**	H	H	OH	*O*-α-l-rhamnopyranosyl
**31**	H	H	H	*O*-β-d-galactopyranosyl

Studies with the leaves of *M. hiemalis* Cambess. led to the isolation of 5-hydroxy-6,8-dimethyl-7-methoxyflavanone (**32**), 6,8-dimethyl-5,7-dimethoxyflavanone (**33**) and 2,7-dihydroxy-6,8-dimethyl-5-methoxyflavanone (**34**), together with the chalcones 2ʹ,4ʹ-dihydroxy-3ʹ,5ʹ-dimethyl-4,6ʹ-dimethoxychalcone (**35**), 2ʹ-hydroxy-3ʹ,5ʹ-dimethyl-4ʹ,6ʹ-dimethoxychalcone (**36**) and 2ʹ,6ʹ-dihydroxy-3ʹ,5ʹ-dimethyl-4ʹ-methoxychalcone (**37**) and the isoflavone 7-hydroxy-6,8-dimethyl-5-methoxy-isoflavone (**38**) [55]. *Myrcia hiemalis* was the only species from which chalcones, C-methylflavanones and isoflavones were isolated.

### 6.2. Terpenoids

**Figure 4:**
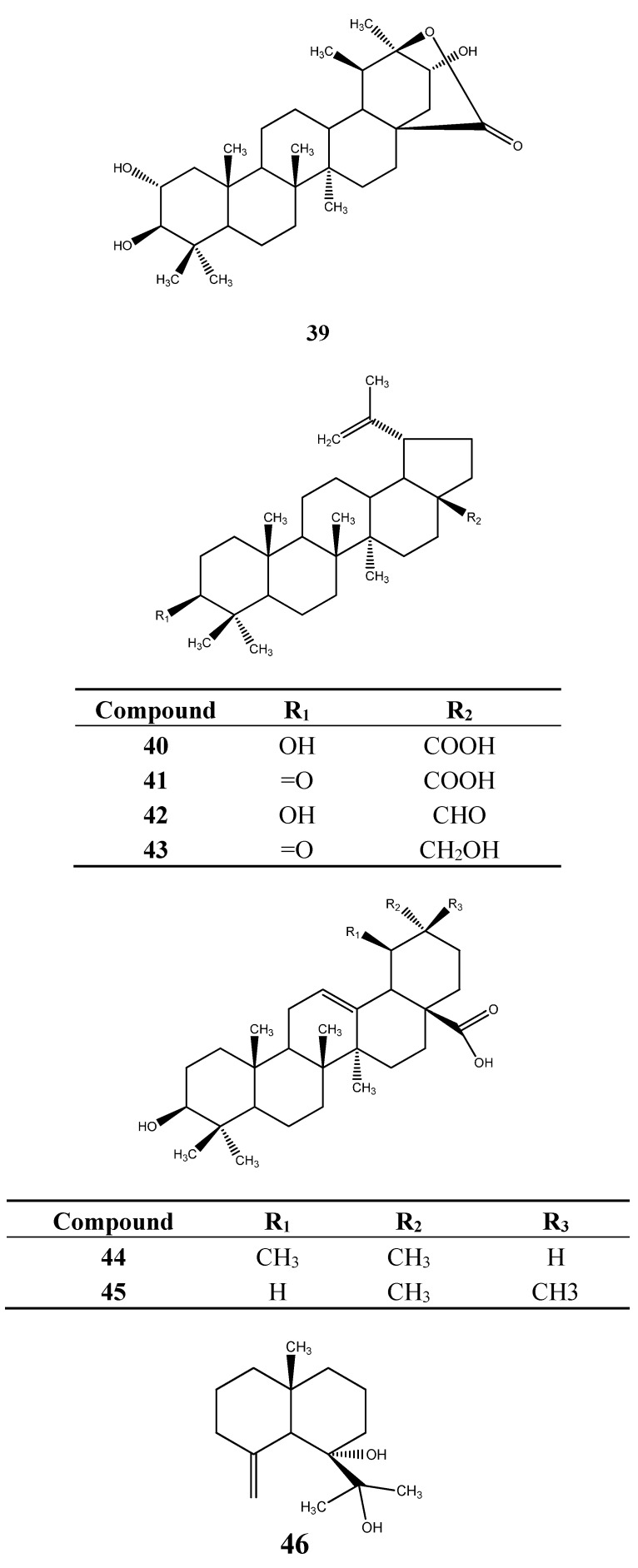

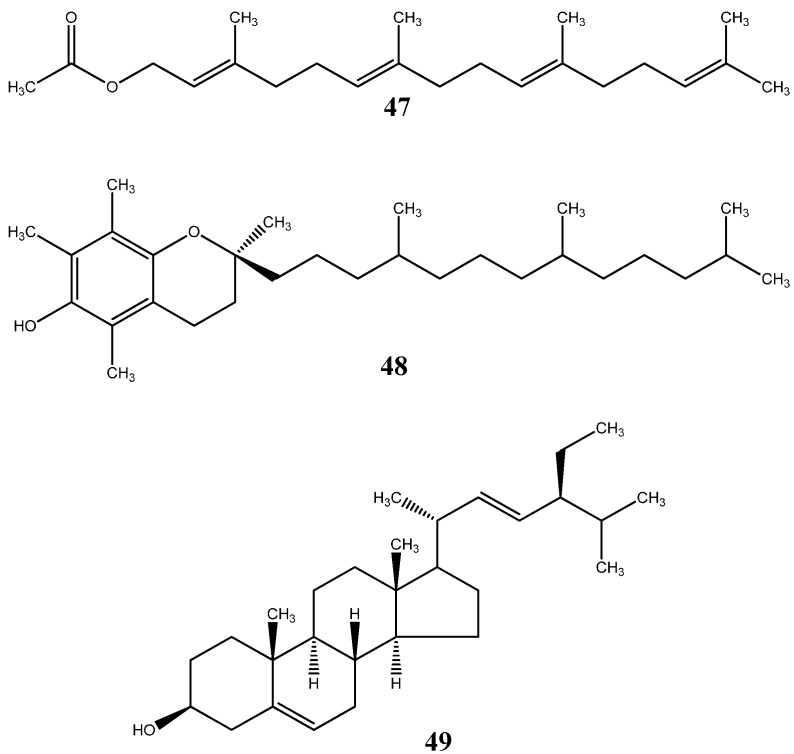
Terpenoids from *Myrcia* species.

Some *Myrcia* species produce terpenoids. 2α,3β,21α-Trihydroxy-28,20β-hydroxytaraxastanolide (**39**) was isolated from *M. hiemalis* [55], and betulinic acid (**40**), betulonic acid (**41**), betulinaldehyde (**42**), betulona (**43**), oleanolic acid (**44**), ursolic acid (**45**) were obtained from *M. myrtillifolia* [55]. The sesqui-, di- and tetraterpenoids eudesm-4-(15)-en-7α,11-diol (**46**) and geranylgeranyl acetate (**47**) and α-tocopherol (**48**), respectively, were also isolated from *M. hiemalis* [55]. Stigmasterol (**49**) was found in *M. myrtillifolia* [55]. Structures of the terpenoids identified from *Myrcia* are in Figure 4.

### 6.3. Organic Acids

**Figure 5:**
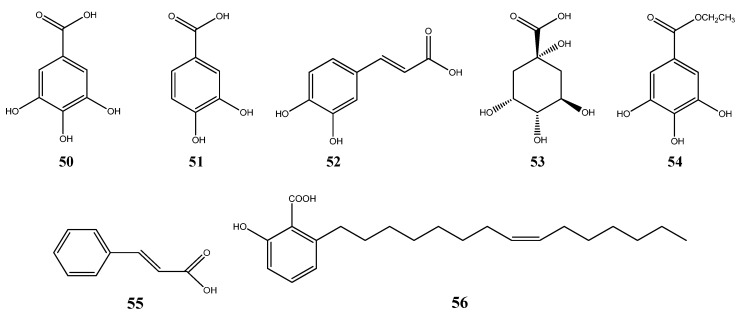
Organic acids isolated from *Myrcia* species.

Some organic acids were isolated from *Myrcia* species (Figure 5). Gallic acid (**50**) was isolated from the leaves of *M. bella* [49] and *M. guianensis* [56]. Protocatechuic acid (**51**) was identified from *M. guianensis* [56] and *M. palustris* [54]. Caffeic acid (**52**), quinic acid (**53**) and the derivative ethyl gallate (**54**) were found in *M. bella* [49]. Cinnamic acid (**55**) was isolated from *M. hiemalis* [55] and ginkgoic acid (**56**) from *M. multiflora* [47].

### 6.4. Acetophenones and Related Compounds

**Figure 6:**
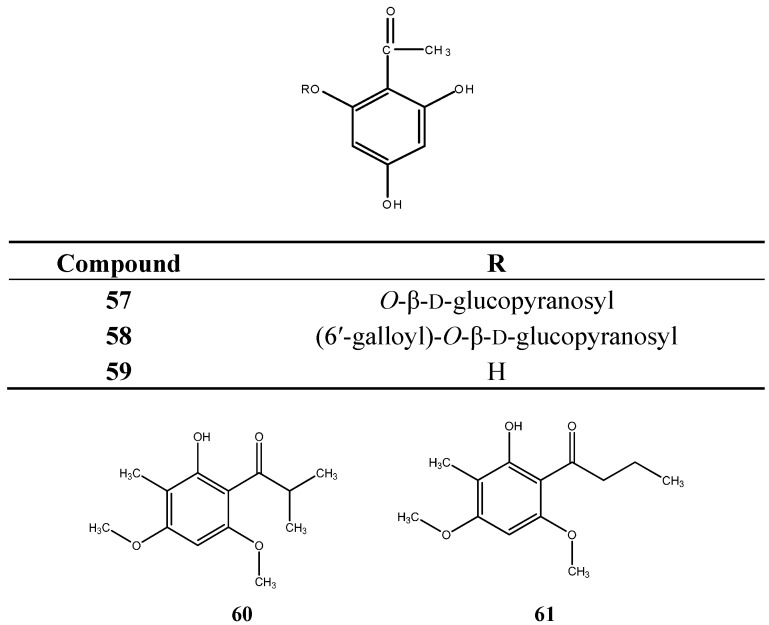
Acetophenones and derivatives from *Myrcia* species.

Myciaphenones A (**57**) and B (**58**) and phloroacetophenone (2ʹ,4ʹ,6ʹ-trihydroxyacetophenone) (**59**), were isolated from *M. multiflora* [47,57]; two derivatives (**60**–**61**) were identified in *M. myrtillifolia* [55]. Structures of compounds **57**–**61** are in Figure 6.

### 6.5. Tannins

**Figure 7:**
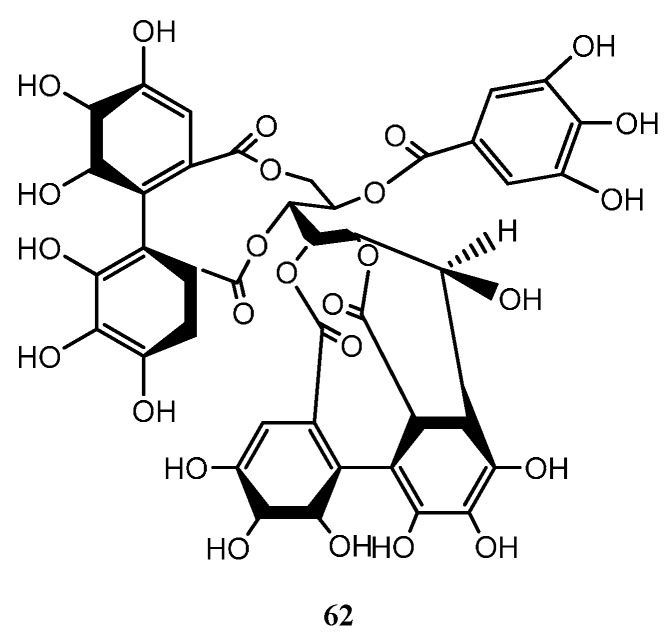

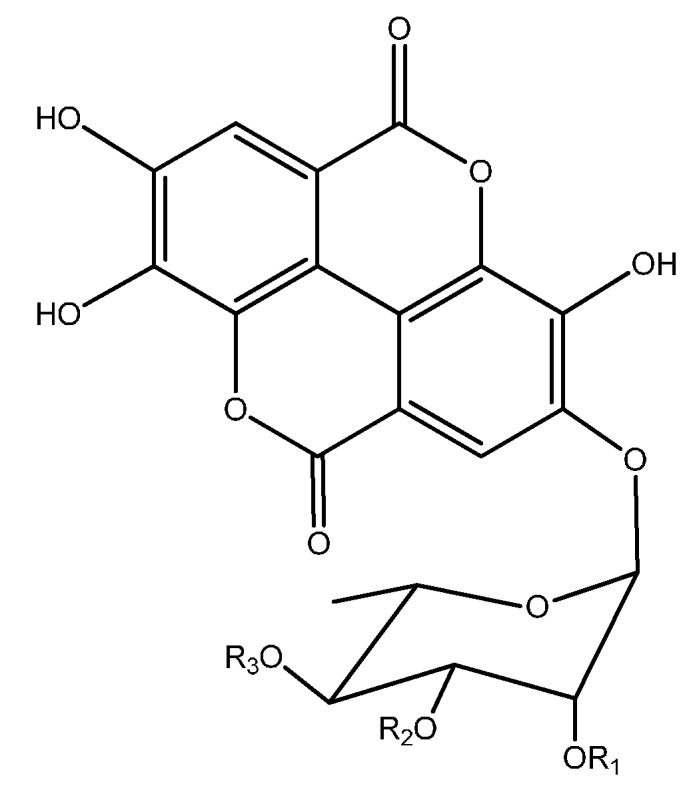
Tannins isolated from *Myrcia* species.

*Myrcia palustris* produced the tannin casuarinin (**62**) together with 4-*O*-(4ʹʹ-*O*-acetyl-α-l-rhamnopyranosyl)-ellagic acid (**63**), 4-*O*-(2ʹʹ,4ʹʹ-*O*-diacetyl-α-l-rhamnopyranosyl)-ellagic acid (**64**), 4-*O*-(2ʹʹ,3ʹʹ-*O*-diacetyl-α-l-rhamnopyranosyl)-ellagic acid (**65**), 4-*O*-(3ʹʹ,4ʹʹ-*O*-diacetyl-α-l-rhamnopyranosyl)-ellagic acid (**66**) and 4-*O*-(2ʹʹ,3ʹʹ,4ʹʹ-*O*-triacetyl-α-l-rhamnopyranosyl)-ellagic acid (**67**) [54]. Structures of the tannins identified from *Myrcia* species are in Figure 7.

**Table:** 

Compound	R_1_	R_2_	R_3_
**63**	H	H	Acetyl
**64**	Acetyl	H	Acetyl
**65**	Acetyl	Acetyl	H
**66**	H	Acetyl	Acetyl
**67**	Acetyl	Acetyl	Acetyl

### 6.6. Alkaloid

**Figure 8:**
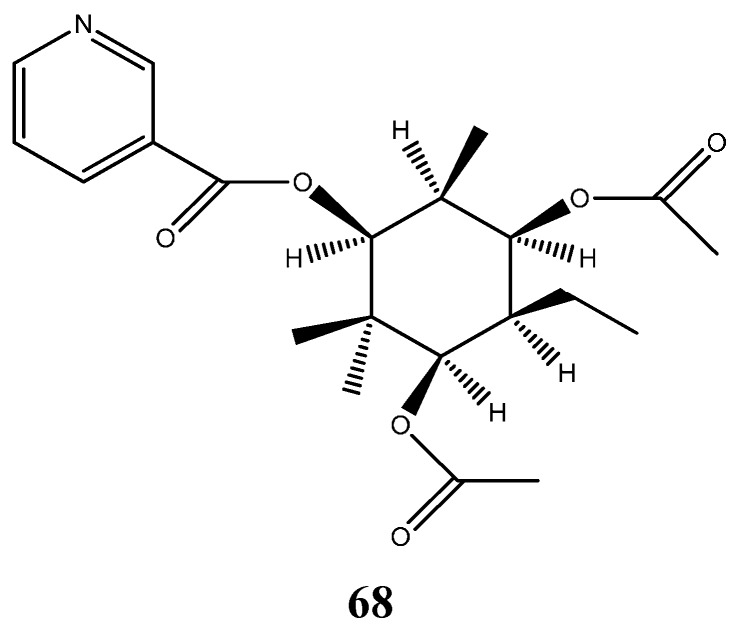
Strucuture of the alkaloid myrciaine.

*Myrcia blanchetiana* (O.Berg) Mattos was the only *Myrcia* species found to produce alkaloid; the nicotinic ester Myrciaine (**68**) was identified from this species [58] (Figure 8).

## 7. Pharmacological Effects of *Myrcia* Extracts and Isolated Compounds

Several studies dealing with biological activities of *Myrcia* extracts and isolated compounds have been published.

### 7.1. Hypoglycemic Potential

The methanol extract and ethyl acetate-soluble portion from *M. multiflora* leaves, one of the species known as “pedra-ume-caá”, showed inhibitory activities on aldose reductase and α-glucosidase, on the increase of serum glucose level on sucrose-loaded rats and on alloxan-induced diabetic mice [47]. The flavanone glucosides myrciacitrins I–II (**1**–**2**), the flavonol glucosides myricitrin (**6**), mearnsitrin (**7**), quercetin (**8**), desmanthin-1 (**9**) and guaijaverin (**10**) and the acetophenone glucoside myrciaphenone B (**58**), all isolated from *M. multiflora*, showed potent inhibitory activity on aldose reductase and α-glucosidase and among them, desmanthin-1 showed the most potent activity on aldose reductase [47]. A further study with the same species confirms potent inhibitory activity on aldose reductase of myrciatrins, including III, IV and V [48]. When comparing the structures of the tested substances, it is observed that desmanthin-1 is the only flavonol glucoside with a galloyl group linked to the sugar unit.

*Myrcia palustris* produced five α-glucosidase inhibitors, the flavonol glucosides myricetin (**13**), quercetin (**20**), casuarinin (**62**), myricetin 3-*O*-β-d-(6ʹʹ-galloyl)-galactopyranoside (**27**) and kaempferol 3-*O*-β-d-galactopyranoside (**31**) [54]. The treatment of mice with the ethanol extract of *M. bella* leaves using 600 mg/kg reduced the fast blood glucose, total cholesterol and triglycerides and it increased hepatic glycogen; the authors conclude that the tested extract has exhibited hypoglycemic properties and possibly acts to regulate glucose uptake by the liver [59]. Although *M. palustris* and *M. bella* are not included in the group as species known as “pedra-ume-caá”, they show hypoglycemic potential.

The number of studies is still small, but it can be observed that flavonol glucosides and acethophenone derivatives are not restricted to species known as “pedra-ume-caá”.

### 7.2. Antiobesity and Mixed Hypolipidemic Effects

Phloroacetophenone (**59**) isolated from *M. multiflora* has hypolipidemic and antiobesity effects related to reduction of triglyceride intestinal absorption and pancreatic lipase activity inhibition [57].

### 7.3. Anti-Hemorrhagic Activity

The aqueous extract and the aqueous residue at 1:1 (*w*/*w*) of the leaves of *M. guianensis* completely inhibited the hemorrhagic effect produced by intradermic injections of crude venom of the snake *Bothrops jararaca* in Swiss mice; the ethyl acetate extract at 1:3 (*w*/*w*) inhibited 90.7%; the *in vitro* venom effect was analyzed by electrophoresis; according to the authors, these observations could explain the traditional use of *M. guianensis* to reduce snake venom effect [18].

### 7.4. Phytotoxic Effect and Allelophatic Potential

The ethyl acetate extract of *M. guianensis* leaves (1% *w*/*v*) showed phytotoxic activity when tested on seed germination and seedlings growth bioassays using the weeds *Mimosa pudica* and *Senna obtusifolia* as test plants; the isolated compounds gallic (**50**) and protocatechuic acids (**51**) showed concentration-dependent allelopathic effects and the strongest activity was observed at 60 ppm [56]. The fractions containing the flavonoids glucosides avicularin (**11**) and juglanin (**12**) isolated from *M. tomentosa* showed potent inhibition of coleoptiles growth using wheat seeds (*Triticum aestivum*) as the test plant [53].

### 7.5. Hepatoprotective Effect

Hydrolysis of phloroacetophenone glucoside (**57**), isolated from *M. multiflora*, gave its aglicone (**59**), which protected mouse liver from injury induced by CCl_4_, probably through its scavenging ability [60]. In the same study, it was observed that the pre-treatment with phloroacetophenone normalized the activities of antioxidant enzymes catalase, glutathione peroxidase, and superoxide dismutase, and increased the levels of reduced glutathione. In addition, it significantly prevented the elevation of serum enzymatic activities, as well as histological alteration.

### 7.6. Antioxidant Effects

The ethanol extracts of *M. laruotteana* and *M. obtecta* leaves showed antioxidant effects in the DPPH assays; these extracts were more active than quercetin [61]. The ethyl acetate and *n*-butanol phases of the hydroalcoholic extract of *M. splendens* and *M. palustris* leaves and stems showed antioxidant activity using DPPH radical and iron reduction assays; myricitrin (**6**) was isolated as the major constituent of the ethyl acetate phase of both species [50].

Studies with *M. rostrata* showed that the content of phenolic compounds (total, tannins, flavonoids) is influenced by environmental factors such as soil micronutrients, rainfall and pH [62], as well as the study with *M. tomentosa* leaves [63].

### 7.7. Others

The aqueous phase of the methanol extract of *M. uniflora* and the isolated flavonoids myricitrin (**6**) and mearnsitrin (**7**) were able to inhibit thyroid peroxidase *in vitro*; according to the authors, the indiscriminate consumption of *M. uniflora* pharmaceutical products (capsules or tinctures for treatment of diabetes mellitus), allied to the nutritional deficiency of iodine, might contribute to the development of hypothyroidism [52].

A US patent dealing with *M. fallax* extracts and its capacity of killing tumor cells derived from human carcinoma of the nasopharynx (KB) was deposited [64].

## 8. Concluding Remarks

*Myrcia* is an economically important genus. It is a rich source of essential oils and it is ornamental. Parts of the plant *in natura*, extracts and capsules of some *Myrcia* species are commercialized as phytomedicine. *Myrcia* species are used in the traditional medicine on the treatment of a variety of illnesses, including diabetes and stomach problems. The essential oils from *Myrcia*, are usually mixtures of sesquiterpenes with small concentrations of monoterpenes. These volatile oils have shown biological activities such as anti-inflammatory, antinociceptive, antimicrobial, among others. Chemical studies with the non-volatile compounds show that *Myrcia* species are a rich source of flavonoids, especially flavonol glucosides; triterpenoids, organic acids and acetophenone derivatives were also isolated from *Myrcia*. Some of the isolated flavonol and flavanone glucosides, their aglicones and acetophenone derivatives show hypoglycemic potential and could explain the traditional use of some of *Myrcia* species to treat diabetes. Acetophenone derivatives are also related to hypolipidemic effects. Therefore, *Myrcia* is a promising source of biologically active compounds. Several species have not been chemically and biologically studied, and others required further studies. As it is observed for other Myrtaceae, the similarity of the species is high and taxonomic and nomenclatural history is complex, resulting in difficulties in identification. Further comparison of chemical composition, biological activities and traditional uses taking into account all species of *Myrcia* s.l. will contribute to the chemosystematic of Myrtaceae.
